# Supplementary material for: SpanSeq: similarity-based sequence data splitting method for improved development and assessment of deep learning projects
Source: NAR Genom Bioinform. 2024 Aug 16;6(3):lqae106. doi: 10.1093/nargab/lqae106 (PMC11327874; doi:10.1093/nargab/lqae106)
Supplement: lqae106_Supplemental_File [file lqae106_supplemental_file.pdf]

SpanSeq: Similarity-based sequence data splitting method for improved development and assessment of deep learning projects

Supplementary Material

Alfred Ferrer Florensa, Jose Juan Almagro Almenteros, Henrik Nielsen, Frank Møller Aarestrup, Philip Thomas Lanken Conradsen Clausen

June 20, 2024

SUPPLEMENTARY FIGURES

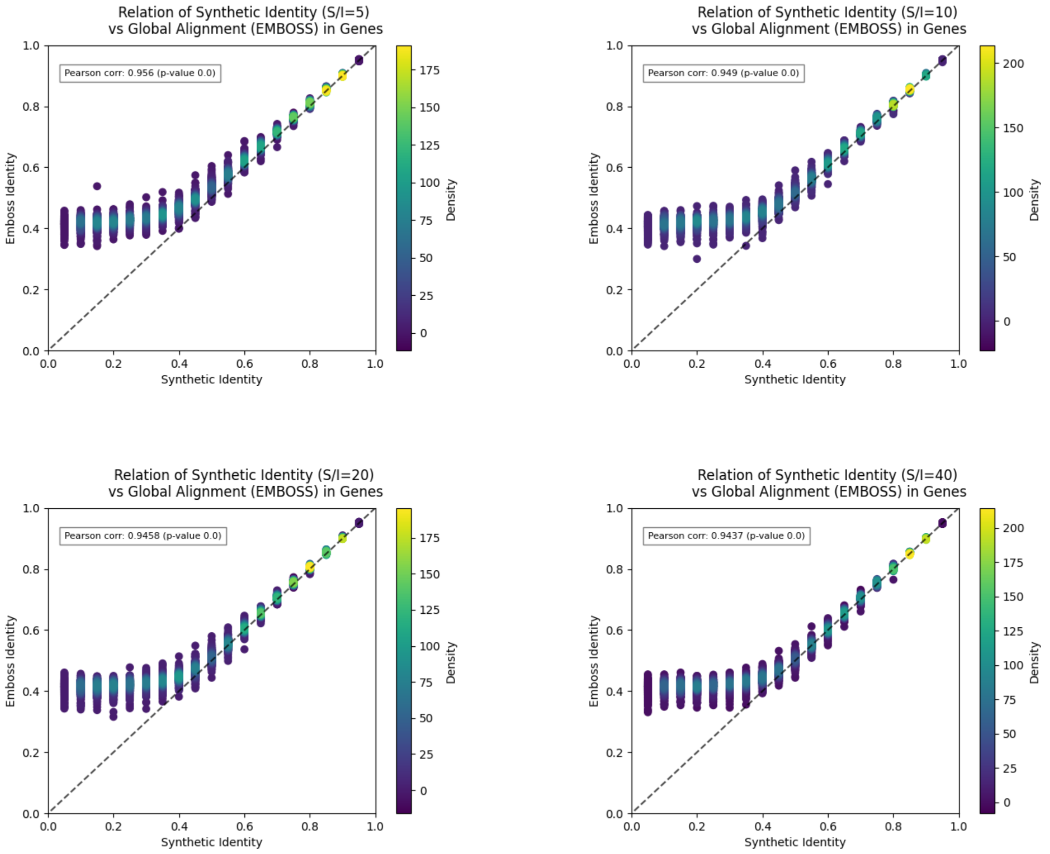

Figure S1: Relation between the identity from the global alignment calculated with EMBOSS [1] and the synthetic identity produced by substitutions and indels (S/I) on genes. The values for the percentage of substitutes and indels have been selected because they can be found in real data.

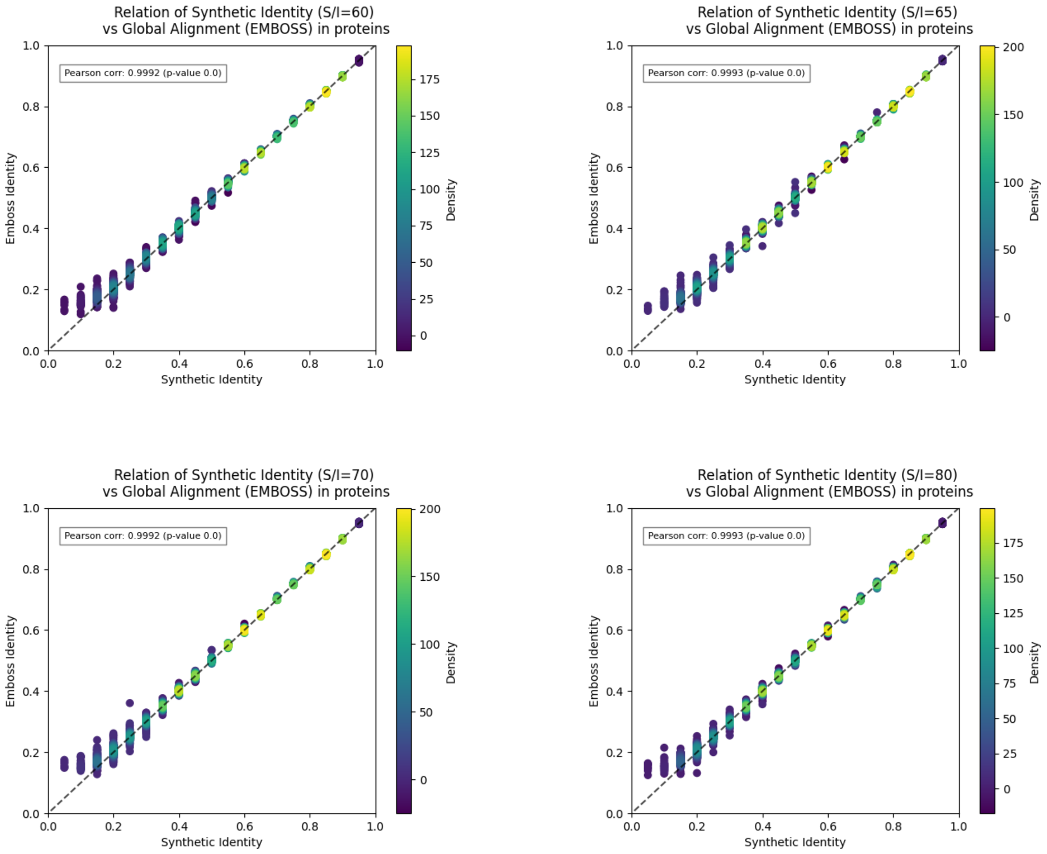

Figure S2: Relation between the identity from the global alignment calculated with EMBOSS [1] and the synthetic identity produced by substitutions and indels (S/I) on proteins. The values for the percentage of substitutes and indels have been selected because they can be found in real data.

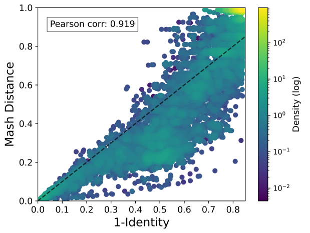

(a) Mash distance against global identity on protein dataset, using k-mer size 6.

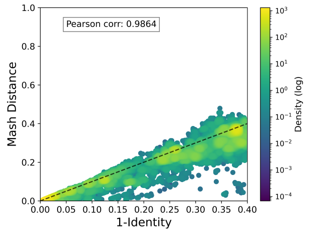

(b) Mash distance against global identity on gene dataset, using k-mer size 8.

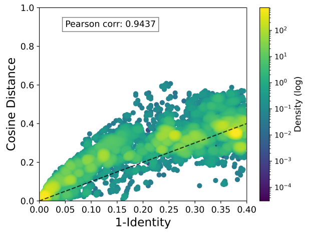

(c) Cosine distance against global identity on gene dataset, using k-mer size 8, minimizer size 5.

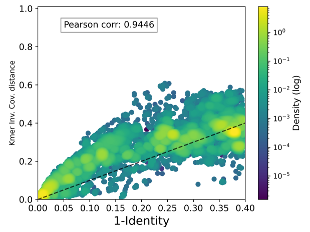

(d) K-mer inverse coverage distance against global identity on gene dataset, using k-mer size 8, minimizer size 5.

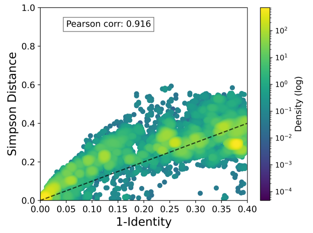

(e) Szymkiewicz–Simpson distance against global identity on gene dataset, using k-mer size 8, minimizer size 5.

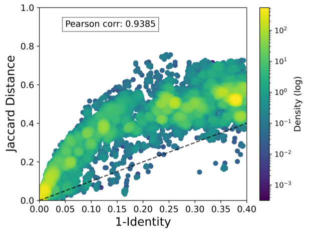

(f) Jaccard distance against global identity on gene dataset, using k-mer size 8, minimizer size 5.

Figure S3: Relation between distance measures available in SpanSeq for amino acids (a) and nucleotides (b-f). The visualization is limited to the identity ranges 100% - 15% for amino acids, and 100% - 60% for nucleotides, due to the artefacts that appear when aligning distant sequences [2] (Supplementary Data, Figures S1 and S2).

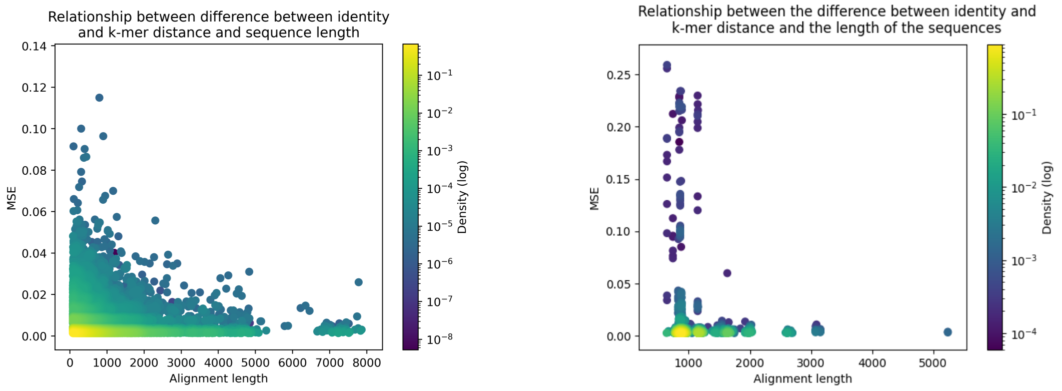

Figure S4: Relationship between length of sequences (which is related to alignment length) and the difference between identity and k-mer distances (MSE), in nucleotides using Cosine Distance (left) and proteins using Mash Distance (right).

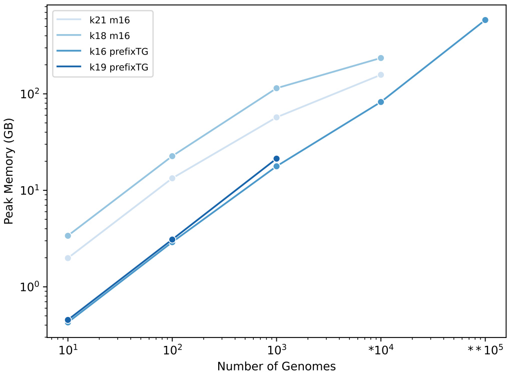

(a) Peak Memory of SpanSeq depending on the amount of samples

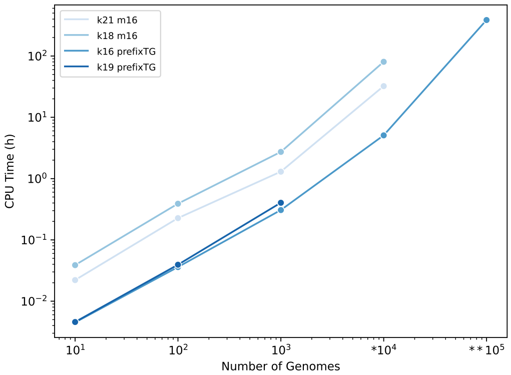

(b) CPU Time of SpanSeq depending on the amount of samples

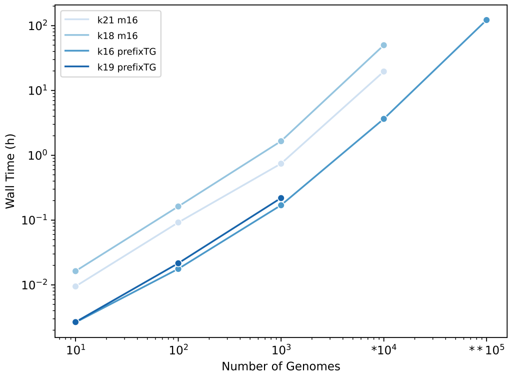

(c) Wall Time of SpanSeq depending on the amount of samples

Figure S5: Performance evaluation of SpanSeq on datasets of bacterial genomes from RefSeq. *Running KMA indexing (step necessary for calculating the distances) with K19 prefixTG has a too big k-mer for running this analysis. **It was only reasonable to run k16 with prefixTG for 10^5.

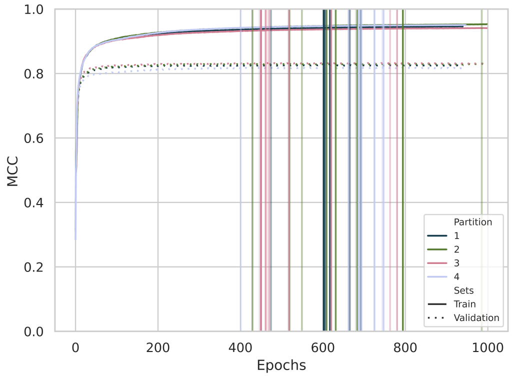

(a) Training process with increased similarity split dataset. The epochs 516, 986, 446, 444 have the best validation set MCC measure (0.83, 0.83, 0.83, 0.82; respectively)

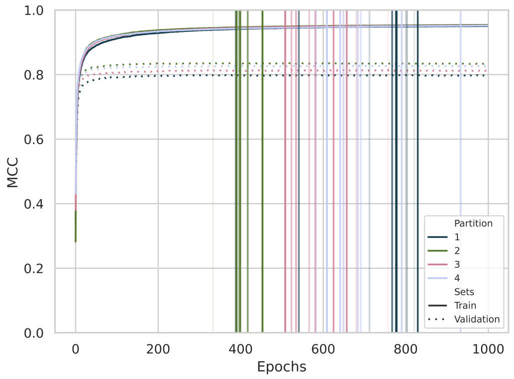

(b) Training process with randomly split dataset. The epochs 821, 333, 757, 657 have the best validation set MCC measure (0.80, 0.83, 0.81, 0.77; respectively)

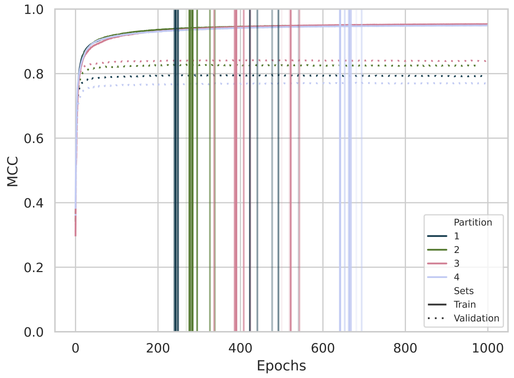

(c) Training process with SpanSeq (cosine) split dataset. The epochs 476, 269, 550, 657 have the best validation set MCC measure (0.62, 0.60, 0.64, 0.62; respectively)

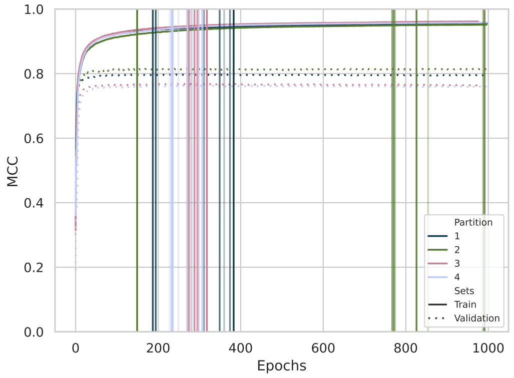

(d) Training process with SpanSeq (GGSearch36) split dataset. The epochs 476, 269, 500, 660 have the best validation set MCC measure (0.79, 0.81, 0.76, 0.76; respectively)

Figure S6: DL-RNA training curves for the different dataset splits. The dashed and continuous lines show the MCC measures of the train and validation sets respectively. The vertical lines show the ten best validation MCC values for each validation set (the transparency of these lines is inversely related to the validation MCC value).

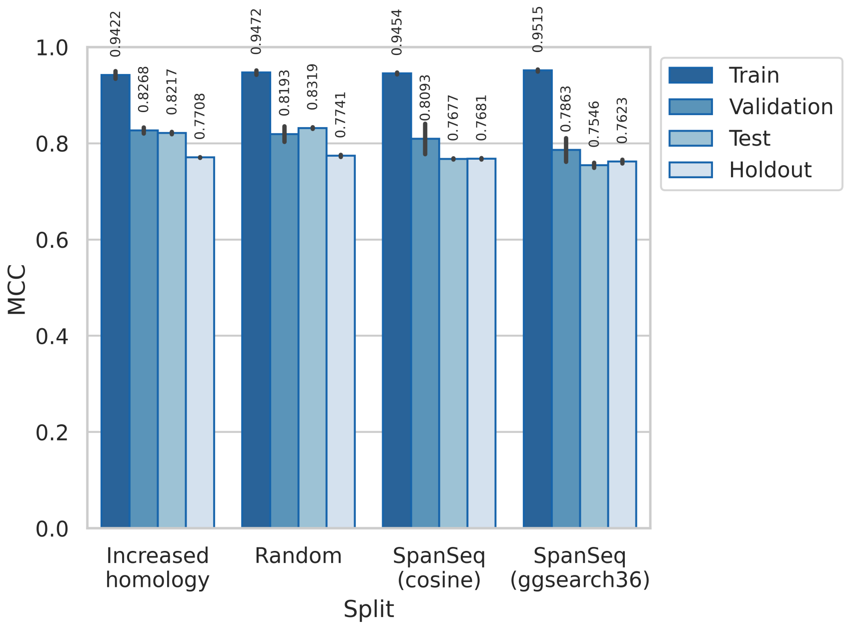

Figure S7: MCC values on the different partitions of data depending on the split method

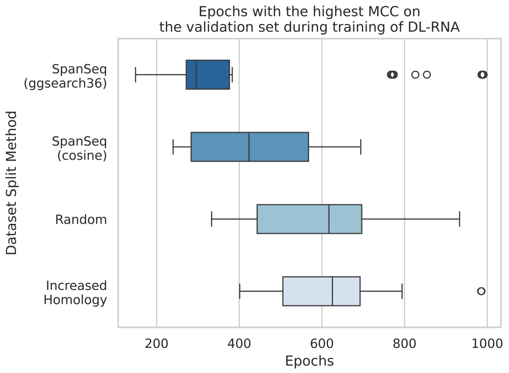

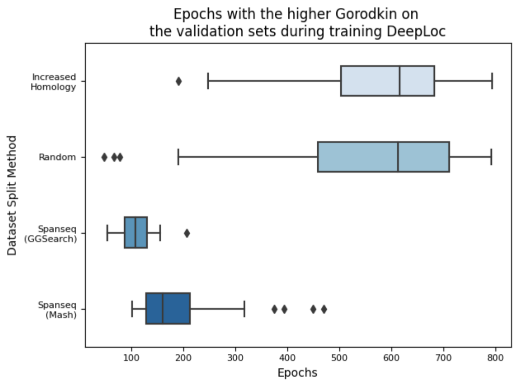

Figure S8: Boxplot showing the best 10 epochs for each of the neural networks (4) for each of the split techniques. On the left, DL-RNA model with MCC used as measure; DeepLoc model with Gorodkin used as the measure.

SUPPLEMENTARY TABLES

	**Increased Similarity**	**Random**	**SpanSeq (Mash)**	**SpanSeq (Alignment)**
Gorodkin value	0.698300	0.653506	0.612709	0.614149
Batch Size	16	64	96	96
Attention Size	256	64	484	64
Clip	3	5	9	9
Convolutional Kernels	5,1,1,21,29,1	21,1,1,9,3,1	1,3,29,1,1,21	1,1,21,1,1,9
Dropouts	0.25,0.1	0.1,0.25	0.5,0.1	0.5,0.25
Learning Rate	0.0005	0.001	0.001	0.001
Number of Filters	10	20	10	10
Number of Features	50	30	50	20
Hidden Feed-forward layer size	484	256	128	384

Table S1: Highest Gorodkin value in the validation set and the hyperparameters selected during the hyperparameter selection.

**Species Clustered**
Wolbachia endosymbiont of Drosophila santomea,<br>Wolbachia endosymbiont of Drosophila melanogaster,<br>Wolbachia pipientis,<br>Wolbachia endosymbiont of Aedes aegypti,<br>Wolbachia endosymbiont of Drosophila simulans,<br>Wolbachia endosymbiont of Drosophila innubila,<br>Wolbachia endosymbiont (group A) of Coremacera marginat
Brucella melitensis,<br>Brucella suis,<br>Brucella abortus,<br>Brucella ceti, Brucella canis,<br>Brucella pinnipedialis
Rhizobium sp. N1341,<br>Rhizobium esperanzae,<br>Rhizobium sp. N113,<br>Rhizobium sp. N621

Table S2: Top 3 clusters with more species by using SpanSeq on the 10^4 genomes from RefSeq and SpanSeq with cosine disance of 0.4.

SUPPLEMENTARY FORMULAS

Distances

$$\textbf{\textit{Cosine Distance}} = D_C(A,B) = 1 - S_C(A,B) := 1 - \cos(\theta) = 1 - \frac{A \cdot B}{\|A\| \cdot \|B\|} \tag{S1}$$

$$\textbf{\textit{Jaccard Distance}} = D_J(A,B) = 1 - S_J(A,B) = 1 - \frac{|A \cap B|}{|A| + |B| - |A \cap B|} \tag{S2}$$

$$\textbf{\textit{Inverse Coverage}} = D_I(A,B) = 1 - S_I(A,B) = 1 - 2\frac{|A \cap B|}{\|A\| + \|B\|} \tag{S3}$$

$$\textbf{\textit{Szymkiewicz–Simpson Distance}} = D_S(A,B) = 1 - S_S(A,B) = 1 - \frac{|A \cap B|}{\min(|A|, |B|)} \tag{S4}$$

Measures

$$\textbf{\textit{MCC}} = \frac{tp \times tn - fp \times fn}{\sqrt{(tp - fp)(tp - fn)(tn - fp)(tn - fn)}} \tag{S5}$$

where:

tp = True Positives
tn = True Negatives
fp = False Positives
fn = False Negatives

$$\textbf{\textit{Gorodkin measure}} = \frac{c \times s - \sum_k^K p_k \times t_k}{\sqrt{(s^2 - \sum_k^K p_k^2) - (s^2 - \sum_k^K t_k^2)}} \tag{S6}$$

where:

$t_k = \sum_i^K C_{ik}$ (the number of times class k truly occurred)
$p_k = \sum_i^K C_{ki}$ (the number of times class k was predicted)
$c = \sum_k^K C_{kk}$ (the total number of samples correctly predicted)
$p_k = \sum_i^K \sum_j^K C_{ij}$ (the totla number of samples)

References

[1] Peter Rice, Ian Longden, and Alan Bleasby. Emboss: the European molecular biology open software suite. *Trends in genetics*, 16(6):276–277, 2000.

[2] Burkhard Rost. Twilight zone of protein sequence alignments. *Protein engineering*, 12(2):85–94, 1999.
